# The ROAM/EORTC-1308 trial: Radiation versus Observation following surgical resection of Atypical Meningioma: study protocol for a randomised controlled trial

**DOI:** 10.1186/s13063-015-1040-3

**Published:** 2015-11-14

**Authors:** Michael D. Jenkinson, Mohsen Javadpour, Brian J. Haylock, Bridget Young, Helen Gillard, Jacqui Vinten, Helen Bulbeck, Kumar Das, Michael Farrell, Seamus Looby, Helen Hickey, Mattheus Preusser, Conor L. Mallucci, Dyfrig Hughes, Carrol Gamble, Damien C. Weber

**Affiliations:** Department of Neurosurgery, The Walton Centre NHS Foundation Trust, Liverpool, L9 7LJ UK; Neuropsychology, The Walton Centre NHS Foundation Trust, Liverpool, L9 7LJ UK; Neuroradiology, The Walton Centre NHS Foundation Trust, Liverpool, L9 7LJ UK; Department of Neurosurgery, Beaumont Hospital, Dublin 9, Ireland; Neuropathology, Beaumont Hospital, Dublin 9, Ireland; Neuroradiology, Beaumont Hospital, Dublin 9, Ireland; Institute of Translational Medicine, University of Liverpool, Liverpool, L69 7BE UK; Institute of Psychology Health and Society, University of Liverpool, Liverpool, L69 7BE UK; Clinical Trials Research Centre, University of Liverpool, Liverpool, L69 7BE UK; Department of Clinical Oncology, Clatterbridge Cancer Centre, Wirral, CH63 4JY UK; brainstrust, Isle of Wight, PO31 7QG UK; Department of Medicine, Comprehensive Cancer Center Vienna, CNS Unit, Medical University of Vienna, A-1090 Vienna, Austria; Department of Paediatric Neurosurgery, Alder Hey Children’s Hospital, Liverpool, L12 2AP UK; Centre for Health Economics and Medicines Evaluation, Bangor University, Bangor, LL57 1UT UK; Centre for Proton Therapy, Paul Scherrer Institute, Villigen, Switzerland

**Keywords:** Atypical meningioma, Radiotherapy, Survival, Outcome

## Abstract

**Background:**

Atypical meningiomas are an intermediate grade brain tumour with a recurrence rate of 39–58 %. It is not known whether early adjuvant radiotherapy reduces the risk of tumour recurrence and whether the potential side-effects are justified. An alternative management strategy is to perform active monitoring with magnetic resonance imaging (MRI) and to treat at recurrence. There are no randomised controlled trials comparing these two approaches.

**Methods/Design:**

A total of 190 patients will be recruited from neurosurgical/neuro-oncology centres across the United Kingdom, Ireland and mainland Europe. Adult patients undergoing gross total resection of intracranial atypical meningioma are eligible. Patients with multiple meningioma, optic nerve sheath meningioma, previous intracranial tumour, previous cranial radiotherapy and neurofibromatosis will be excluded. Informed consent will be obtained from patients. This is a two-stage trial (both stages will run in parallel):

Stage 1 (qualitative study) is designed to maximise patient and clinician acceptability, thereby optimising recruitment and retention. Patients wishing to continue will proceed to randomisation.

Stage 2 (randomisation) patients will be randomised to receive either early adjuvant radiotherapy for 6 weeks (60 Gy in 30 fractions) or active monitoring.

The primary outcome measure is time to MRI evidence of tumour recurrence (progression-free survival (PFS)). Secondary outcome measures include assessing the toxicity of the radiotherapy, the quality of life, neurocognitive function, time to second line treatment, time to death (overall survival (OS)) and incremental cost per quality-adjusted life year (QALY) gained.

**Discussion:**

ROAM/EORTC-1308 is the first multi-centre randomised controlled trial designed to determine whether early adjuvant radiotherapy reduces the risk of tumour recurrence following complete surgical resection of atypical meningioma. The results of this study will be used to inform current neurosurgery and neuro-oncology practice worldwide.

**Trial registration:**

ISRCTN71502099 on 19 May 2014.

## Background

Meningiomas arise from the linings of the brain, account for 25 to 33 % of adult primary brain tumours and have a peak incidence at age 40 to 60 years [1]. The World Health Organisation (WHO) [1] classifies three grades:

1. Benign (grade I) meningioma (approximately 90 %)
2. Atypical (grade II) meningioma (approximately 7 %)
3. Anaplastic (grade III) meningioma (approximately 3 %)

**Fig. 1:**
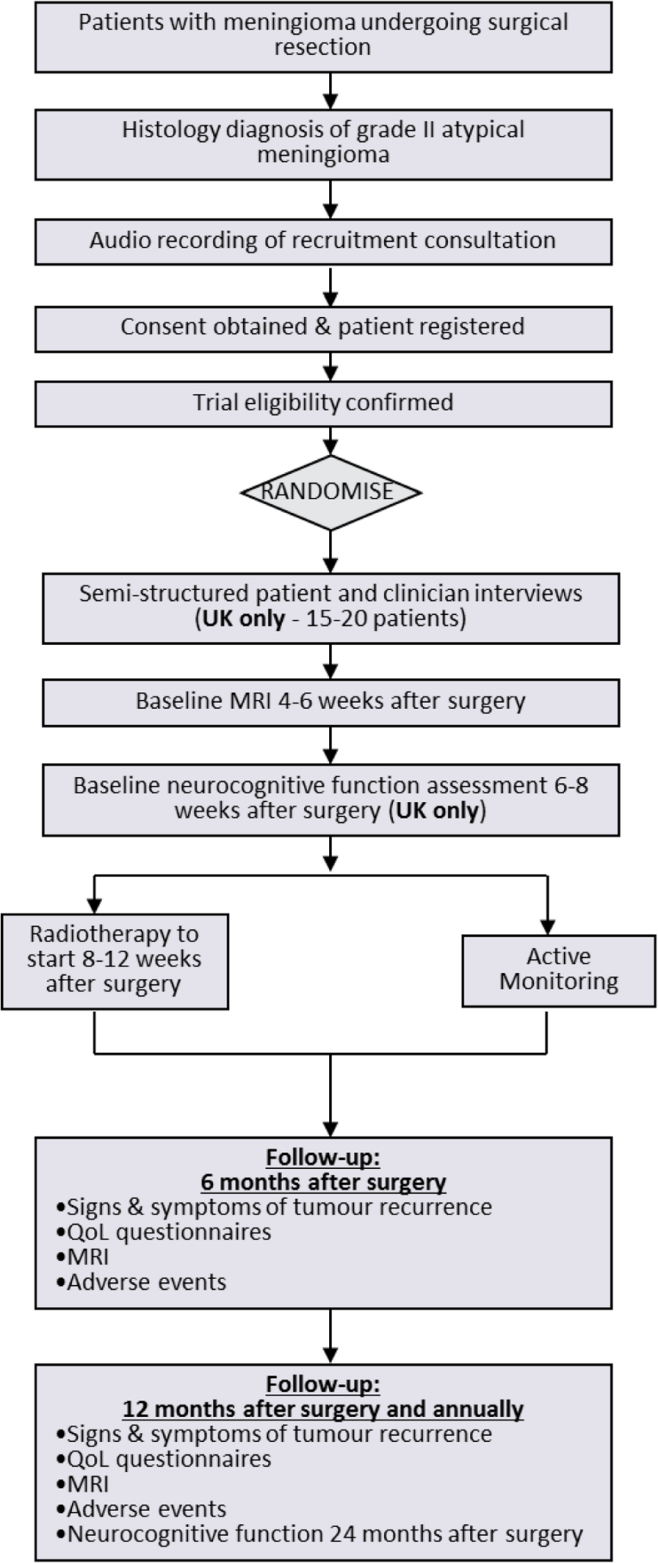
ROAM/EORTC 1308 study design

The annual UK incidence of atypical meningioma is estimated at 0.2 to 0.5/100,000 per year and approximately 150 undergo surgical resection each year. Since the publication of the 2000 WHO classification, the reported incidence of atypical meningioma has risen to 20 to 35 % [2–4]; nevertheless, they remain uncommon. The primary treatment for symptomatic or enlarging atypical meningioma is surgical excision, and the completeness of the resection is an important prognostic factor [5]. Simpson defined the extent of resection into five categories [5]:

- Simpson 1: complete tumour removal, including dural attachment and any abnormal bone
- Simpson 2: complete tumour removal, with coagulation of dural attachment
- Simpson 3: complete tumour removal, without resection or coagulation of its dural attachment
- Simpson 4: partial tumour removal
- Simpson 5: biopsy only

In modern neurosurgery Simpson 1–3 constitute gross total resection (GTR), and Simpson 4–5 constitutes subtotal resection (STR).

Benign (grade I) meningiomas have a low risk of recurrence (approximately 10 % at 5 years) following surgical resection and are managed by active monitoring with magnetic resonance imaging (MRI) scans. For anaplastic (grade III) meningioma, adjuvant radiotherapy is indicated following surgery to prolong time to recurrence; however, the 5-year progression-free survival is only approximately 10 % [6]. In atypical meningioma (grade II), the 5-year tumour recurrence rates are reported as between 39 and 58 %, and in those patients with residual solid tumour, radiotherapy is administered to reduce the risk of recurrence. However, in patients with gross total resection the role of early adjuvant radiotherapy has not been defined, and the options of radiotherapy or active monitoring are discussed with individual patients. Whilst radiotherapy has been shown to be an effective adjuvant treatment in some studies [7, 8] but not others [2, 9, 10], no consensus exists as to which of these approaches is best. A recently published systematic review concluded that since atypical meningioma preferentially recur within 5 years, future studies should investigate the role of early adjuvant radiotherapy in these patients [11]. There have been no randomised controlled trials in this tumour population. Currently, the treatment decision for adjuvant radiotherapy varies according to the patient, surgeon and neuro-oncologist preference [12, 13], and some European expert opinion recommends that all atypical meningioma patients should have radiotherapy (http://meningiomauk.org/radiotherapy/). No agreement exists on the current standard of care for patients with atypical meningioma undergoing complete resection. Whilst the use of radiotherapy may obviate the need for further surgical procedures, this must be balanced against the potential risks of radiotherapy (from 3.4 to 16.7 % [11]), which include neurocognitive impairment, hypopituitarism and radiation-induced tumours. Equally, tumour recurrence can also affect neurocognitive function (NCF) and quality of life (QoL). Tumours that recur can be treated with further surgery and radiotherapy.

ROAM/EORTC-1308 (Radiotherapy versus Observation following surgical resection of Atypical Meningioma) was funded to determine whether early adjuvant radiotherapy reduces tumour recurrence compared to active monitoring in patients with newly diagnosed atypical meningioma who have undergone gross total resection.

### Primary objective

The primary objective of the study was to determine whether early adjuvant fractionated radiotherapy reduces the risk of tumour recurrence compared to active monitoring in newly diagnosed atypical meningioma.

### Secondary objectives

The secondary objectives were as follows:

1. To assess the early and late effects of fractionated radiotherapy
2. To assess and compare quality of life in patients with atypical meningioma according to the treatment arm
3. To assess and compare the neurocognitive function in patients with atypical meningioma according to treatment arm
4. To record the second-line treatments (surgery, radiotherapy, and radiosurgery) used at tumour recurrence according to the treatment arm
5. To determine the overall survival (OS) at 5 years
6. To assess the cost-effectiveness of adjuvant radiotherapy compared to active monitoring (UK sites only)
7. To correlate proliferation rate and molecular characteristics with time to tumour recurrence (separate research funding will be sought).

## Methods/Design

### Design overview

ROAM/EORTC-1308 is an international, multi-centre, phase III, randomized controlled trial comparing early adjuvant radiotherapy (intervention) with observation (comparator) in patients who have undergone gross total resection of an intracranial atypical meningioma. This is a two-stage trial, and both stages will run in parallel:

#### Stage 1 (qualitative study)

The stage is designed to maximize patient and clinician acceptability, thereby optimizing recruitment and retention. Patient permission will be sought to allow audio-recording of the consultations during which patients are approached for recruitment to the trial. The qualitative study will examine how information about the trial is exchanged by clinicians and patients as they discuss ROAM and consent is sought. As well as audio-recording the recruitment consultations, a qualitative researcher will subsequently interview a sub-sample of patients and clinicians. The qualitative study will draw on previously described methods [14–16] to identify the source of any recruitment difficulties and design bespoke strategies to optimise recruitment. This approach has demonstrated success in enhancing recruitment in previous trials [17] and aims to improve the patient experience by improving information exchange and communication. Specifically, the qualitative study will compare discussions during recruitment consultations, with clinicians’ and patients’ interpretations of these consultations.

#### Stage 2 (randomization)

In stage 2, patients will be randomized in a 1:1 ratio to early adjuvant radiotherapy (60 Gy in 30 fractions) for 6 weeks (intervention) or active monitoring with MRI (comparator).

### Research setting

The trial will be conducted across 20 adult regional neurosurgery units in the UK and Ireland. As part of an intergroup collaboration with the European Organisation for Research and Treatment of Cancer (EORTC) the trial will also open in 22 centres in mainland Europe. A feasibility survey completed by 10 UK centres provided information on the number of eligible patients and confirmed that the sample size is achievable.

### Neurocognitive function assessment

NCF will be assessed using a standard validated battery of tests to measure verbal and visual memory, executive skills, processing speed, language, working memory, mood and visuo-spatial construction. These are important parameters to assess and determine whether radiotherapy has any adverse impact on neurocognitive function in patients with meningioma. The following tests will be used: Hopkins Verbal Learning Test, REY Complex Figure Test, Dass21, Stroop Trail Making Test, Symbol Digit Modality Test, WAIS-IV (Digit Span and Blocks Test), Graded Naming Test and Benton Verbal Fluency Test. Additionally, consent will be sought for longer term follow up NCF assessment 5 years after surgery to assess the later effects of treatment in both the radiotherapy and observation arms.

### Patient and public involvement

The involvement of consumers has been fundamental to the design of this trial, and it is recognised that patients and carers have unique experience and expertise, which they can bring to this study. This enables them to be part of the solution to the problems faced by researchers when designing a trial. They have played an active role in the idea generation, trial design and funding application. Their voice will continue to play a significant role in the preparation and execution of this study and the dissemination of results.

### Funding and ethics approval

The ROAM trial is funded by a £1.36 million grant from the National Institute of Health Research Health Technology Assessment (NIHR-HTA) programme (project number 12/173/14). MDJ is the chief investigator and MJ is the co-chief investigator. The study protocol, patient information sheets and consent forms received ethical approval from the North East Newcastle and North Tyneside 2 Research Ethics Committee (ref: 15/NE/0013). Additionally, this trial will be funded by a €230,000 grant from the Brain Tumour and Radiation Oncology groups of the EORTC, which will facilitate the trial opening in mainland Europe. DCW is the lead for the intergroup collaboration with the EORTC.

### Study population

The trial will be open to all adult patients with atypical meningioma who meet the eligibility criteria.

### Inclusion criteria

For inclusion, each patient must meet the following criteria:

1. Histologically confirmed newly diagnosed solitary atypical meningioma (WHO grade II) based on the 2007 WHO criteria [1]
2. Age ≥ 16 years
3. All anatomical locations allowed except optic nerve sheath tumour
4. Complete resection (Simpson 1, 2 or 3) as assessed by the surgeon
5. Able to commence radiotherapy between within 12 weeks of surgery (ideally 8 to 12 weeks)
6. WHO performance status 0, 1 or 2
7. Women of reproductive potential must use effective contraception for the whole duration of the treatment
8. Absence of any psychological, familial, sociological or geographical condition potentially hampering compliance with the study protocol and follow-up schedule; those conditions should be discussed with the patient before registration in the trial.

### Exclusion criteria

Patients exhibiting any of the following will be excluded from the study:

1. Neurofibromatosis type II (NF-2)
2. Optic nerve sheath tumours
3. Multiple meningiomas
4. Radiation induced meningioma
5. Clinical evidence of second malignancies, except a history of cervix carcinoma in situ and/or basal cell carcinoma
6. Previous intracranial tumour
7. Pregnant or lactating women.

### Primary endpoint

The primary endpoint will be the time to MRI evidence of tumour recurrence or death due to any cause (disease-free survival (DFS))

### Secondary endpoints

The secondary endpoints will include the following:

1. Toxicity assessed by CTCAE (Common Terminology Criteria for Adverse Events)
2. Quality of life
3. Neurocognitive function
4. Time to second-line (salvage) treatment (surgery, radiotherapy, and radiosurgery)
5. Time to death (overall survival (OS))
6. Health economic analysis (incremental cost per QALY gained)

**Disease-free survival (DFS)** will be counted from the date of surgery until the date of MRI evidence of tumour recurrence or death due to any cause. Only clear dural thickening at the distinction of the investigator is to be considered tumour.

**Overall survival (OS)** will be counted from the date of surgery until death due to any cause.

### Randomisation

Patients will be randomised to early radiotherapy or observation in a ratio of 1:1. Written informed consent will be obtained following surgical resection and histopathological confirmation of an atypical meningioma.

### Proposed sample size

Atypical meningioma 5-year tumour recurrence rates are reported as between 39 and 58 %. A 0.05 level two-sided log-rank test for equality of survival curves with 80 % power would require 86 patients in each arm (total number of events required = 46) to detect an absolute reduction from 40 % in the control group arm to 20 %. A strong magnitude of effect is required to impact clinical practice and establish a treatment policy across the NHS in the UK. This is due in part to the expense of radiotherapy but also the burden to patients - due to the side effects of radiotherapy (hair loss, skin irritation, cognitive decline, and secondary malignancy) and its delivery requiring patients to attend the hospital daily (Monday through Friday) for 6 weeks. Patient retention will be high as patients with atypical meningioma are routinely followed up for the long term, and data will be collected at routine clinic visits. However, an adjustment to allow for a 10 % loss to follow-up has been made, requiring a total of 190 patients to be recruited. This sample size calculation has been agreed upon with the EORTC. The UK arm of the trial will aim to deliver 118 participants with a total of 29 events providing 60 % power. The remaining participants would be recruited across Europe within the EORTC intergroup collaboration.

### Statistical analysis

The trial will be analysed and reported using the ‘Consolidated Standard of Reporting Trials’ (CONSORT) and the International Conference on Harmonisation E9 guidelines. A full and detailed statistical analysis plan will be developed prior to the final analysis of the trial. The main features of the statistical analysis plan are included here.

The primary analysis will be by intention-to-treat principle, as far as is practically possible. Results will be presented throughout using 95 % confidence intervals and a 5 % level of statistical significance. Time to event outcomes will be analysed using Kaplan Meier curves, log rank tests and Cox Proportional Hazards models. Assumptions of proportional hazards will be investigated. Ordinal categorical outcomes will be analysed using an ordinal logistic model. Continuous outcomes will be assessed using ANCOVA methods.

### Heath economic analysis

There are no existing economic studies of treatment options in atypical meningioma, and to assess the balance of the potential benefits of reduced recurrence rates against the costs, we will conduct a cost-utility analysis from the perspective of the NHS. Resource use will be based on entries made in designated sections of patients' case report forms, Hospital Episode Statistics data sourced from the Health and Social Care Information Centre for patients recruited in England, and data from the hospital Patient Administration Systems as indicated below:

1. The CRF will be used to record data on procedures and interventions as well as dates of patient transfers both within and between hospitals from admission to discharge.
2. Six months after randomising the last patient at each recruiting centre, the Finance departments of each centre will be contacted, and a request submitted for Ward name, ward speciality, the average cost per bed day on the ward, and the financial year to which the costs refer. The Information Technology or Patient Administration Departments of each centre will also be contacted, and a request will be submitted (via the CTRC, to maintain patient anonymity) for the Patient NHS Numbers (or some other means of linking the patient to the trial), ward name, ward speciality (if possible), start date on the ward, end date on the ward, and number of occupied bed days on the ward.
3. Data on Hospital Episode Statistics (HES) from the beginning of the financial year prior to baseline, to 5 years follow-up will be accessed centrally via biennial downloads from the Health and Social Care Information Centre.

Unit costs will be obtained from NHS reference costs. The number of QALY gained will be estimated by administering the EQ-5D-5 L and applying a UK tariff for generating utilities. An economic (Markov) model will be specified with appropriate health states to project lifetime costs and consequences. Costs and QALYs occurring after the first year will be discounted at 3.5 % per annum. Incremental cost-effectiveness ratios will be compared with threshold values, and the joint uncertainty in costs and benefits considered (in the trial-based analysis) through the application of bootstrapping and (in the model) using probabilistic sensitivity analysis to generate cost-effectiveness acceptability curves.

### Translational research studies

The authors have an established brain tumour biobank (Walton Research Tissue Bank- WRTB; North Wales REC No 11/WNo03/2). Consent will be sought from patients for tumour tissue and serum banking, use of samples for future research projects including genetics studies, and for collaboration with academic and commercial partners. Tumour tissue (paraffin-embedded and snap-frozen, if available) from surgery will be sent to the WRTB. Serum samples will be taken when the patient undergoes each MRI scan and sent to the WRTB. Future translational research themes will include the following:

1. MRI - Volumetric measurements will be used to determine tumour recurrence. The effects of radiotherapy on normal brain adjacent to the resection cavity will be studied.
2. Tumour biology - to investigate whether genetic, epigenetic or biochemical factors explain individual variation in tumour recurrence and response to radiotherapy.
3. Serum analysis - to investigate biomarkers of tumour recurrence.

### Dissemination of results

The communication and dissemination strategy will actively involve participating centres, their staff and service users and the professional bodies involved (for example, Society of British Neurological Surgeons and the EORTC) and relevant charitable organisations (including brainstrust, The Brain Tumour Charity, and Brain Tumour Research). Communication and dissemination of results will be assisted by members of the study team, including using social networking sites. Findings of the trial will also be presented at National and International meetings of relevant professional bodies and research groups. The results of the trial will be published in peer-reviewed journals.

## Discussion

This protocol describes the design of a randomised controlled trial to evaluate the clinical effectiveness and cost-effectiveness of early adjuvant radiotherapy for reducing tumour recurrence in patients undergoing gross total resection of atypical meningioma. This is the first randomised controlled to compare early adjuvant radiotherapy with active monitoring. This study will inform clinical practice and generate a high quality tumour and serum biobank in a cohort of atypical meningioma patients.

## Trial status

The trial is scheduled to open in 2015 (http://www.nets.nihr.ac.uk/projects/hta/1217314).

## Abbreviations

CNS: central nervous system
CRF: case report form
DFS: disease-free survival
EORTC: European Organisation for Research and Treatment of Cancer
GTR: gross total resection
IMRT: intensity modulated radiation therapy
MRI: magnetic resonance imaging
NCF: neurocognitive function
NHS: National Health Service
OS: overall survival
QALY: quality-adjusted life year
RCT: randomised controlled trial
STR: subtotal resection
WHO: World Health Organisation
